# Atypical Chemokine Receptor 3 “Senses” CXC Chemokine Receptor 4 Activation Through GPCR Kinase Phosphorylation[Fn fn7]

**DOI:** 10.1124/molpharm.123.000710

**Published:** 2023-10

**Authors:** Christopher T. Schafer, Qiuyan Chen, John J. G. Tesmer, Tracy M. Handel

**Affiliations:** Skaggs School of Pharmacy and Pharmaceutical Sciences, University of California, San Diego, La Jolla, California (C.T.S., T.M.H.) and Departments of Biological Sciences and of Medicinal Chemistry and Molecular Pharmacology, Purdue University, West Lafayette, Indiana (Q.C., J.J.G.T.)

## Abstract

Atypical chemokine receptor 3 (ACKR3) is an arrestin-biased receptor that regulates extracellular chemokine levels through scavenging. The scavenging process restricts the availability of the chemokine agonist CXCL12 for the G protein-coupled receptor (GPCR) CXCR4 and requires phosphorylation of the ACKR3 C-terminus by GPCR kinases (GRKs). ACKR3 is phosphorylated by GRK2 and GRK5, but the mechanisms by which these kinases regulate the receptor are unresolved. Here we determined that GRK5 phosphorylation of ACKR3 results in more efficient chemokine scavenging and *β*-arrestin recruitment than phosphorylation by GRK2 in HEK293 cells. However, co-activation of CXCR4-enhanced ACKR3 phosphorylation by GRK2 through the liberation of G*βγ*, an accessory protein required for efficient GRK2 activity. The results suggest that ACKR3 “senses” CXCR4 activation through a GRK2-dependent crosstalk mechanism, which enables CXCR4 to influence the efficiency of CXCL12 scavenging and *β*-arrestin recruitment to ACKR3. Surprisingly, we also found that despite the requirement for phosphorylation and the fact that most ligands promote *β*-arrestin recruitment, *β*-arrestins are dispensable for ACKR3 internalization and scavenging, suggesting a yet-to-be-determined function for these adapter proteins. Since ACKR3 is also a receptor for CXCL11 and opioid peptides, these data suggest that such crosstalk may also be operative in cells with CXCR3 and opioid receptor co-expression. Additionally, kinase-mediated receptor cross-regulation may be relevant to other atypical and G protein–coupled receptors that share common ligands.

**SIGNIFICANCE STATEMENT:**

The atypical receptor ACKR3 indirectly regulates CXCR4-mediated cell migration by scavenging their shared agonist CXCL12. Here, we show that scavenging and *β*-arrestin recruitment by ACKR3 are primarily dependent on phosphorylation by GRK5. However, we also show that CXCR4 co-activation enhances the contribution of GRK2 by liberating G*βγ*. This phosphorylation crosstalk may represent a common feedback mechanism between atypical and G protein–coupled receptors with shared ligands for regulating the efficiency of scavenging or other atypical receptor functions.

## Graphical abstract

**Figure:**
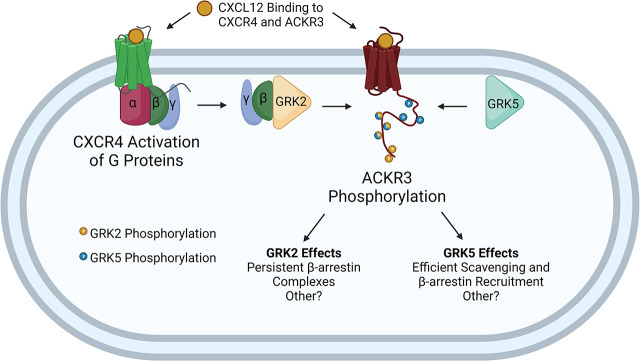

## Introduction

Chemokine receptors mediate cell migration in the context of immune system function, development, and disease by responding to small (8–14 kDa) protein agonists (chemokines) and activating G protein signaling cascades that lead to cell motility (Kufareva et al., 2017). Cell positioning also depends on the establishment of localized chemokine gradients, which provide directional cues for migrating cells (Boldajipour et al., 2008; Griffith et al., 2014). Thus, in addition to G protein–coupled canonical chemokine receptors (CCKRs) that directly mediate cell movement, a subclass of G protein–independent atypical chemokine receptors (ACKRs) indirectly contribute to migration by controlling extracellular chemokine concentrations and shaping chemokine gradients through scavenging. Scavenging by ACKRs restricts CCKR access to chemokines and regulates canonical receptor downregulation, thereby maintaining chemokine receptor responsiveness and ability to promote cell migration (Nibbs and Graham, 2013; Vacchini et al., 2016).

ACKR3 (also known as CXCR7) is an atypical receptor that shares the endogenous ligands of the CCKRs: CXCL11 (an agonist of CXCR3) and CXCL12 (the sole agonist of CXCR4) (Balabanian et al., 2005; Burns et al., 2006; Thelen and Thelen 2008). Although the receptor has been reported to function on its own (Rajagopal et al., 2010;Lipfert et al., 2013), ACKR3 also cooperates with CXCR3 and CXCR4, in part by scavenging CXCL11 and CXCL12 (Burns et al., 2006). Scavenging involves internalization of receptor-bound chemokines into the cell for degradation, followed by recycling of the receptor back to the cell surface for further rounds of chemokine consumption (Burns et al., 2006; Thelen and Thelen, 2008). The importance of ACKR3 is underscored by the fact that its absence leads to severe defects in CXCR4-mediated interneuron migration (Saaber et al., 2019) and development of the zebrafish lateral line primordium (Valentin et al., 2007).

Like other ACKRs, ACKR3 does not activate heterotrimeric G proteins (Hoffmann et al., 2012; Saaber et al., 2019), with the exception of cell-specific coupling in astrocytes (Odemis et al., 2012; Fumagalli et al., 2020). However, it is phosphorylated by GPCR kinases (GRKs), which results in the recruitment of *β*-arrestins (Rajagopal et al., 2010; Hoffmann et al., 2012; Saaber et al., 2019; Zarca et al., 2021). In some studies, *β*-arrestin2 has been reported to be required for scavenging (Luker et al., 2010; Vacchini et al., 2016), yet others show efficient chemokine uptake in cells lacking *β*-arrestins (Montpas et al., 2018; Saaber et al., 2019). Nevertheless, phosphorylation by GRKs seems critical for ACKR3 scavenging (Saaber et al., 2019; Zarca et al., 2021). C-terminal phosphorylation is increased upon CXCL12 stimulation of ACRK3 and switches the receptor from ubiquitin-mediated lysosomal degradation toward plasma membrane recycling (Lau et al., 2020; Wong et al., 2020).

Most studies have implicated GRK2 in the internalization of ACKR3 as well as ACKR3-mediated arrestin recruitment and chemokine scavenging (Saaber et al., 2019; Zarca et al., 2021), though others suggest little impact of GRK2 (Sarma et al., 2022). Although GRK5 also phosphorylates ACKR3 (Saaber et al., 2019), the importance of GRK5 and the relative contribution and precise roles of GRK2 versus GRK5 have not been elucidated. Here we compare the impact of GRK2 and GRK5 on ACKR3 internalization, *β*-arrestin recruitment, and scavenging. Although published studies have focused on GRK2 as the key ACKR3 kinase in HEK293 cells and primary neurons (Saaber et al., 2019; Zarca et al., 2021), our results suggest that GRK5 dominates ACKR3 phosphorylation in HEK293A cells. However, we also find that phosphorylation of ACKR3 by GRK2 is increased by co-activation of CXCR4. The data are consistent with the dependence of GRK2 on G*βγ* (Pitcher et al., 1992), which is liberated by CXCL12 stimulation of CXCR4 but not by ACKR3 and with the G*βγ* independence of GRK5 (Pronin et al., 1998). These results suggest a kinase-mediated mechanism that enables ACKR3 to sense the activation of CXCR4 and respond in a cell-specific manner depending on the co-expression and activation of the canonical GPCR.

## Materials and Methods

### Materials

Unless otherwise stated, all chemicals and reagents were purchased from Sigma and Fisher. HEK293A WT, HEK293 WT, and CRISPR GRK and *β*-arrestin1/2-knockout cells were generous gifts from Aska Inoue (Tohoku University) (Pandey et al., 2021; Kawakami et al., 2022). Methoxy e-Coelenterazine (Prolume Purple) was purchased from Nanolight Technologies (Prolume LTD), and fluorescent antibodies from Li-COR Biosciences. CXCL12 ELISA kits were from R&D Systems.

### Cloning

N-terminally FLAG-tagged human ACKR3 (residues 2–362) was cloned into pcDNA3.1 expression vector either alone (FLAG_ACKR3) or followed by a C-terminal fusion of *Renilla* luciferase II (ACKR3_RlucII) in the pcDNA3.1 vector for use in bioluminescence resonance energy transfer (BRET) assays. For purified receptor studies, ACKR3 (residues 2–362) with an N-terminal HA signal sequence was cloned into pFasBac vector, followed by C-terminal 10His and FLAG tags. Site-directed mutagenesis was performed by overlap extension and confirmed by Sanger sequencing.

### CXCL12 Purification from *E. Coli*

The chemokine CXCL12 was expressed and purified as previously described (Gustavsson et al., 2017). Briefly, a mature chemokine sequence, preceded by an 8His tag and an enterokinase cleavage site, was cloned into a pET21-based vector and expressed in BL21(DE3)pLysS cells by isopropyl *β*-d-thiogalactopyranoside (IPTG) induction. Cells were lysed by sonication, and chemokine-containing inclusion bodies were dissolved in 50 mM Tris, 6 M guanidine-HCl, 50 mM NaCl, pH 8.0. The chemokines were bound to a Ni-NTA column, washed with 50 mM MES, 6 M guanidine-HCl, 50 mM NaCl, pH 6, and eluted with 50 mM acetate, 6 M guanidine-HCl, 50 mM NaCl, pH 4. CXCL12 was refolded in 50 mM Tris, 500 mM arginine-HCl, 1 mM EDTA, 1 mM glutathione disulfide, pH 7.5, before removal of the tag by enterokinase. The cleaved material was then bound to a C18 HPLC column (Vydac) (buffer A: 0.1% trifluoroacetic acid; buffer B: 0.1% trifluoroacetic acid, 90% acetonitrile) and eluted by a linear gradient of buffer B from 33% to 45%. The peak was collected, lyophilized, and stored at −80°C until use.

### Arrestin Expression and Purification

Expression and purification of *β*-arrestin1/2 was described previously (Vishnivetskiy et al., 2014). Briefly, the pTrcHisB plasmid containing bovine *β*-arrestin1 and *β*-arrestin2 (1–393) were transformed into *E. coli* Rosetta cells, and protein expression was induced with the addition of 25 *μ*M (*β*-arrestin1) and 35 *μ*M IPTG (*β*-arrestin2) for 4 hours at 30°C. Cells were collected by centrifugation, and the pellet was processed immediately or stored at −80°C. The cell pellets were resuspended and homogenized in 20 mM MOPS pH 7.5, 200 mM NaCl, 5 mM EDTA, 2 mM dithiothreitol (DTT), 1 mM phenylmethylsulfonyl fluoride (PMSF), and leupeptin, lima bean trypsin protease inhibitor. Cells were lysed using an Avestin C3 emulsifier. The lysate was clarified by centrifugation at 18,000 × g for 60 minutes. The supernatant was collected, and arrestin was precipitated by the addition of (NH_4_)_2_SO_4_ to a final concentration 0.32 mg/ml. Precipitated arrestin was collected by centrifugation at 18,000 × g for 90 minutes and dissolved in a buffer containing 20 mM MOPS (pH 7.5), 2 mM EDTA, and 1 mM DTT and then centrifuged again at 18,000 × g for 60 minutes to remove insoluble parts. The supernatant containing soluble arrestin was applied onto a heparin column and eluted with a linear NaCl gradient (0.2–1 M). Fractions containing arrestin were identified by SDS-PAGE and combined. For *β*-arrestin1, the salt concentration of the pooled fractions was adjusted to 50 mM, loaded onto a 5 ml HiTrap Q HP column (Cytiva), and eluted with a linear NaCl gradient. For *β*-arrestin2, the salt concentration of the pooled fractions was adjusted to 100 mM, and the solution was loaded onto a linked 1 ml HiTrap Q HP and 1 ml HiTrap SP HP column. *β*-arrestin2 flows through the Q column but binds the SP column. The columns were uncoupled, and a linear NaCl gradient was used to elute *β*-arrestin2 from the SP column. The fractions containing arrestin were identified by SDS-PAGE and combined, concentrated with a 30 kDa cutoff Amico concentrator to ∼500 *μ*l, and then further purified using a Superdex 200 increase 10/300 GL column (Cytiva) equilibrated with 20 mM MOPS (pH 7.5), 150 mM NaCl, and 0.5 mM Tris (2-carboxyethyl) phosphine (TCEP). The peak fractions were collected, concentrated with a 30 kDa cutoff Amicon concentrator, and stored at −80°C.

### GRK Purification

GRK2 and GRK5 expression and purification were described previously (Sterne-Marr et al., 2013; Beyett et al., 2020). Briefly, a pMAL plasmid containing human full-length GRK5 with C terminal 6his tag was transformed into *E. coli* Rosetta cells. The expression of GRK5 was induced by the addition of 200 *μ*M IPTG at OD around 0.6 to 0.8, and the cultures were incubated with shaking at 18°C overnight. For purification cell pellets were resuspended and homogenized in lysis buffer [20 mM HEPES pH 8.0, 400 mM NaCl, 0.1% Triton-X (v/v), 2 mM DTT, DNase, 0.1 mM PMSF and leupeptin, lima bean trypsin protease inhibitor]. The cells were then lysed using an Avestin C3 emulsifier and centrifuged at 18,000 rpm for 30 minutes. The supernatant was combined and loaded onto a 3 ml home-packed Ni^2+^-NTA column pre-equilibrated with buffer A (20 mM HEPES pH 8.0, 400 mM NaCl, 0.5 mM DTT). The column was then washed with 50 ml buffer A, followed by 100 ml buffer B (20 mM HEPES pH 8.0, 100 mM NaCl, 0.5 mM DTT plus 20 mM imidazole). The bound protein was eluted in approximately 2 ml fractions with buffer B plus 200 mM imidazole. The purity of GRK5-HIS after this step was about 60% as revealed by Coomassie blue staining of samples assessed via SDS–PAGE. The fractions were pooled and loaded onto a linked 1 ml HiTrap Q HP and 1 ml HiTrap SP HP column. GRK5-HIS flows through the Q column and binds to the SP column. The columns were uncoupled, and a linear NaCl gradient (0.1–0.6 M) was used to elute GRK5-HIS from the SP column. GRK5 elutes with approximately 0.3 to 0.5 M NaCl. The fractions containing GRK5-HIS were identified by SDS-PAGE and combined, concentrated with a 50 kDa cutoff Amicon concentrator to ∼500 *μ*l, and then further purified using a Superdex 200 increase 10/300 GL column equilibrated with 20 mM HEPES pH 8.0, 100 mM NaCl, and 0.5 mM TCEP. The peak fractions were collected, concentrated with a 50 kDa cutoff Amicon concentrator, and stored at −80°C.

Human GRK2 S670A with a C-terminal hexahistidine tag was expressed in *Spodoptera frugiperda* (*Sf*9) cells using the Bac-to-Bac insect cell expression system (Life Technologies). The insect cells were harvested 48 hours postinfection and homogenized with buffer containing 20 mM HEPES pH 8.0, 400 mM NaCl, 2 mM DTT, 1 mM PMSF, and leupeptin, lima bean trypsin protease inhibitor. Cells were lysed using an Avestin C3 emulsifier. The lysate was clarified by centrifugation at 18,000 × g for 60 minutes. GRK2 was purified from the clarified lysate as previously described for GRK5 using nickel-nitrilotriacetic acid affinity chromatography. The purity of GRK2-HIS after this step was ∼90%, as revealed by Coomassie blue staining of samples assessed via SDS–PAGE. Fractions containing GRK2 were pooled and further purified on a Superdex 200 increase 10/300 GL column equilibrated with 20 mM HEPES pH 8.0, 100 mM NaCl, and 0.5 mM TCEP. The peak fractions were collected, concentrated with a 50 kDa cutoff Amicon concentrator, and stored at −80°C.

### ACKR3 Expression and Purification from *Sf*9 Cells

Expression and purification of ACKR3 from *Sf*9 cells were performed as previously described (Gustavsson et al., 2016; Yen et al., 2022). Briefly, S*f*9 cells were transfected with ACKR3 or CXCL12_LRHQ_, a high-affinity variant of CXCL12 with residues 1 to 3 replaced by the motif LRHQ, in pFasbBac vectors using X-tremeGene transfection reagent (Roche) to produce baculovirus. The receptors and CXCL12_LRHQ_ were co-expressed by infecting S*f*9 cells at a density 2 × 10^6^ cells/ml with a multiplicity of infection of 6 for each virus. After 48 hours, cell pellets were collected and stored at −80°C. Membranes were prepared by dounce-homogenization in hypotonic buffer (10 mM HEPES pH 7.5, 10 mM MgCl_2_, 20 mM KCl) and then three times with hypotonic buffer with 1 M NaCl. Between each cycle of douncing, the membranes were pelleted by centrifugation at 50,000 × g for 30 minutes and resuspended. The prepared membranes were then solubilized in 50 mM HEPES pH 7.5, 400 mM NaCl, 0.75%/0.15% dodecyl maltoside/cholesteryl hemisuccinate with 2 mg/ml iodoacetamide and a Protease Inhibitor tablet (Roche). After 4 hours, the insoluble material was removed by 50,000 × g centrifugation for 30 minutes, and the supernatant was added to Talon resin (Clontech) with 20 mM imidazole to bind overnight at 4°C. The resin was transferred to a column and washed with WB1 [50 mM HEPES pH 7.5, 400 mM NaCl, 0.1/0.02% lauryl maltose neopentyl glycol (LMNG)/cholesteryl hemisuccinate (CHS), 10% glycerol, 20 mM imidazole), followed by WB2 (WB1 with 0.025/0.005% LMNG/CHS), and finally eluted with elution buffer (WB2 with 250 mM imidazole). The elutions were pooled and concentrated to 500 *µ*l before passing over a PD MiniTrap G-25 desalting column (GE Healthcare) equilibrated with 50 mM HEPES pH 7.5, 100 mM NaCl, 0.025/0.005% LMNG/CHS, 10% glycerol. The final protein concentration was calculated using an A_280_ extinction coefficient of 85,000, snap-frozen in liquid nitrogen, and stored at −80°C until use.

### In Vitro Pulldown of Arrestin by ACKR3

To phosphorylate the receptors, 1.1 *µ*M of purified ACKR3:LRHQ complex was incubated with 1.1 *µ*M CXCL12_LRHQ_ and either 1.1 *µ*M of GRK2 or GRK5 in 50 mM HEPES pH 8.0, 10 mM MgCl_2_, 0.1% LMNG, 0.005% 1,2-dioctanoyl-sn-glycero-3-phospho-1'-myo-inositol-4’,5′-bisphosphate (Avanti) with 1 mM ATP for 20 minutes at room temperature. Next, purified *β*-arrestin1 or 2 was added to a final concentration of 2.2 *µ*M and allowed to complex for 40 minutes at room temperature. M2 anti-FLAG-resin (5 *µ*l) (Sigma) was then added to the reaction and incubated at 4°C for 1 hour. The bound complexes were washed in batch 3× with 50 mM HEPES pH 7.5 150 mM NaCl, 0.01/0.001% LMNG/CHS and eluted by adding 250 *µ*g/ml 3×FLAG peptide (final concentration, Sigma). The total supernatant was analyzed via 10% SDS-PAGE. Band densities were quantified using ImageJ software, and the amount of arrestin pulled down was reported as a percentage of the amount of ACKR3 in the same experiment after normalizing by molecular weight.

### In Vitro ACKR3 Mass Spectrometry

Phosphosite mapping was performed at the Purdue University Proteomics Facility. Briefly, ACKR3 in LMNG or nanodisc was first phosphorylated by GRK2 and GRK5 and then digested with trypsin. The fragments were analyzed via high-resolution mass spectroscopy without TiO_2_ enrichment, and phosphorylation sites were identified through peptide ionization patterns compared with the non-phosphorylated primary amino acid sequence.

### Arrestin Binding to ACKR3 Measured by BRET

Recruitment of *β*-arrestin2 to ACKR3 and CCR2 was measured with a BRET2 assay as previously described (Gustavsson et al., 2019; Yen et al., 2022). HEK293A cells were initially plated at 750,000 cells/well in six-well plates in Dulbecco’s modified Eagle’s media (DMEM) supplemented with 10% FBS and GlutaMax (Gibco) with 5% CO_2_. The following day, the cells were transfected with 100 ng ACKR3_RlucII DNA (or FLAG_CCR2_rlucII) and 2 *µ*g GFP10_*β*-arrestin2 (GFP_*β*arr2, a gift from N. Heveker, Université de Montréal, Canada) both in pcDNA3.1, with 400 ng empty pcDNA3.1 vector using the TransIT-LT1 transfection system (MirusBio) per manufacturer’s protocol and expressed for 40 hours. For experiments with CXCR4, G*βγ*, or GRK3-C-terminus (GRK3-CT) co-expressions, transfections were augmented with either 500 ng CXCR4 DNA, 1 *µ*g GRK3-CT (Bovine, residues 547–688; gift from N. Lambert, Augusta University) DNA, or 500 ng each of G*β*_1_ and G*γ*_2_ DNA (gift from A. Inoue, Tohoku University) in pcDNA3.1 vector, and the amount of ACKR3_RlucII and GFP_*β*arr2 DNA was reduced to 50 ng and 1 *µ*g, respectively, to accommodate the additional DNA. On the day of the experiment, the cells were washed with PBS (137 mM NaCl, 2.7 mM KCl, 10 mM Na_2_HPO_4_, 1.8 mM KH_2_PO_4_, pH 7.4) and mechanically lifted with Tyrode’s Buffer (25 mM HEPES, 140 mM NaCl, 1 mM CaCl_2_, 2.7 mM KCl, 12 mM NaHCO_3_, 5.6 mM glucose, 0.5 mM MgCl_2_, 0.37 mM NaH_2_PO_4_, pH 7.5) and counted on a Vicell cell counter (Beckman Coulter). Next, 100,000 cells were plated in each well of a white, clear bottom 96-well plate (BD Falcon) and allowed to re-adhere for 45 minutes at 37°C. For IT1t-treated samples, the CXCR4 antagonist was added at 100 *µ*M final concentration before re-adhering. Arrestin expression was verified using a Spectramax M5 plate fluorometer (Molecular Devices) with 485 nm excitation, 538 nm emission, and 530 nm cutoff. White backing tape (PerkinElmer) was applied to the plate, and the Prolume Purple luciferase substrate was added to a final concentration of 5 *µ*M. Total luminescence was measured using a VictorX Light multilabel plate reader (PerkinElmer) with no filter, an integration time of 0.1 second. CXCL12 was then added to each well at indicated final concentrations, and the plate was read at 410 nm and 515 nm after 20 minutes of incubation at 37°C. The BRET ratios (515 nm emission/410 nm emission) were baseline matched and normalized to wild-type receptor (WT; E_max_) measured in the same experiment on the same day. The reported data represents a combined data set of three independent experiments tested in duplicate or triplicate. Points were fitted with a sigmoidal dose–response model using SigmaPlot 11.0 (Systat Software Inc.).

The *β*-arrestin2 binding time courses were set up as previously described with the following exceptions. All experiments were read with a TECAN Spark luminometer (Tecan Life Sciences) at 37°C using default BRET2 settings (blue emission 360–440 nm, red emission 505–575 nm) and a 0.5 second integration time. Experiments were read for 5 minutes before 100 nM final concentration CXCL12 was added, and BRET readings were collected for an additional 30 minutes. BRET ratios were calculated by red emission/blue emission, and the chemokine and mock-treated wells were averaged for each experiment. Percent change in BRET due to CXCL12 binding was calculated as the ratio between BRET_CXCL12_ and BRET_Mock_. Data presented represent a combined data set of three independent experiments, each performed in triplicate. The area under curves were calculated using GraphPad Prism 9 (GraphPad Software, Inc.).

### ACKR3 Internalization Measured by BRET

**Fig. 1.**
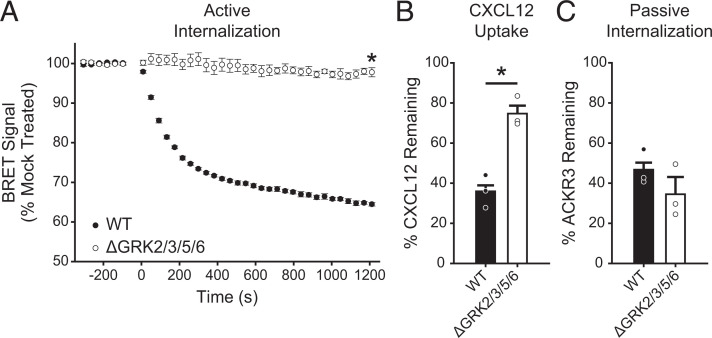
GRKs mediate efficient CXCL12-induced internalization and chemokine scavenging by ACKR3. (A) CXCL12-promoted, active internalization following stimulation with 100 nM chemokine at 37°C was monitored by BRET between ACKR3_RlucII and rGFP_CAAX in WT and ΔGRK2/3/5/6 HEK293A cells. (B) Chemokine uptake by WT and ΔGRK2/3/5/6 cells expressing ACKR3 was detected by quantification of the remaining CXCL12 in the media by ELISA. The extent of scavenging was determined by comparison with cells transfected with empty vector. (C) Constitutive cycling, or passive internalization, of ACKR3 in WT and ΔGRK2/3/5/6 cells was quantified by tracking the loss of “prelabeled” receptor by flow cytometry. Errors are reported as standard deviations, and values are the average of three independent experiments performed in triplicate. Individual experiments are presented as points in B and C. Statistical significance was determined by unpaired *t* test. *P < 0.001.

Agonist-mediated internalization of ACKR3 was measured by BRET2 between ACKR3_RlucII and rGFP_CAAX (a gift from M. Bouvier, Université de Montréal) as previously described (Namkung et al., 2016). Samples were prepared as described for *β*-arrestin2 recruitment time courses, with the exception of the transfected DNA amounts. HEK293 cells were transfected with 42 ng ACKR3_RlucII and 170 ng rGFP_CAAX DNA, with empty pcDNA3.1 to bring the total DNA amount to 2.5 *µ*g/well. Data presented in Figs. 1, 3, and 4C were collected with a PerkinElmer Victor Luminometer, while the time courses in Figs. 4D and 7 were measured on a Tecan Spark luminometer. All settings were identical to those used for arrestin association (previously described). Data are presented as percent change compared to mock-treated wells and are a composite of three independent experiments. The percent changes after 30 minutes were compared for statistical analysis.

### Constitutive ACKR3 Internalization Measured by Flow Cytometry

ACKR3 was stably expressed in HEK293 cells by lentiviral spinoculation and selection with hygromycin (Tiscornia et al., 2006). The stable, homogenous ACKR3 expression was necessary to resolve changes to the surface receptors by flow cytometry. Cells were grown to confluency in 6 cm dishes before washing with cold PBS on ice and lifting with cold Accutase (Innovative Cell Technologies, Inc.). Next, 100,000 cells were transferred to each well of two conical, 96-well plates, one for 37°C experimental samples and the other kept at 4°C as a control. The cells were washed with cold fluorescence-activated cell sorter (FACS) buffer (PBS, 0.5% bovine serum albumin) and labeled with 0.02 *µ*g/well unconjugated anti-ACKR3 antibody (11G8, R&D Systems) for 1 hour at 4°C. Unbound antibody was then washed away with bovine serum albumin buffer. Prewarmed assay buffer (DMEM, 0.5% BSA, 25 mM HEPES pH 7.5) was added to each well of the 37°C plate, and the plate was moved to a 37°C incubator for 45 minutes. The 4°C control plate was left in the 4°C refrigerator during this step. The samples were then washed with FACS buffer and labeled with 1 *µ*l/well of phycoerythrin-conjugated anti-mouse secondary (F0102B, R&D Systems) 1 hour at 4°C. Surface ACKR3 was assessed by flow cytometry using a GuavaCyte benchtop flow cytometer (MilliporeSigma). The geometric mean fluorescence intensity representing the amount of surface labeling for each experiment was quantified using FlowJo software (FlowJo). The constitutive internalization was then represented by the ratio of the geometric mean fluorescence intensity of 37°C samples to the 4°C controls measured on the same day.

### ACKR3 Scavenging of CXCL12 as Determined by ELISA

CXCL12 uptake by ACKR3 was quantified by ELISA per the manufacturer’s protocols (R&D Systems). Briefly, HEK293 cells were seeded on a six-well plate at 750,000 cells/well and transfected the next day with 200 ng of ACKR3_RlucII DNA or empty vector per well as previously described. After 16 hours, the cells were mechanically lifted, counted, and replated at 80,000 cells per well into a 96-well plate and allowed to re-adhere for 6 hours at 37°C or in a 96-well BRET plate and reattached for 30 minutes at 37°C. The BRET plates were washed with Tyrode’s buffer, and Prolume Purple was added to a final concentration of 5 *µ*M. The total ACKR3 expression was determined by luminescence. After 6 hours, the media was exchanged for media containing 25 nM CXCL12 and incubated at 37°C for 16 hours. The media was carefully collected from each well, and cellular debris was removed with a 4-minute spin at 250 × g. The remaining CXCL12 was detected using an R&D Systems ELISA kit and read using a Spectramax M5 plate reader. The amount of chemokine removed was quantified by the ratio of cells expressing ACKR3 to those transfected with empty vectors from the experiments performed on the same day. Three separate experiments were performed in triplicate and averaged together to determine the final amounts of CXCL12 uptake.

### Detection of Extracellular Signal-Regulated Kinase Phosphorylation by Western Blot

Detection of phosphorylation of extracellular signal-regulated kinase (ERK) activation performed as previously described (Saaber et al., 2019). HEK293 cells were grown to confluency in a 24-well plate and transfected with 200 ng FLAG_ACKR3 per well. After 8 hours, the media was removed and replaced with DMEM without FBS for overnight serum starvation. The wells were treated with 10 nM CXCL12 final concentration at the given time points at 37°C. IT1t-treated samples were incubated for 45 minutes with 100 *µ*M IT1t before beginning the CXCL12 additions. Cells were harvested with hot sample buffer (20 mM Tris pH 6.8, 12.5 mM EDTA, 20% glycerol, 1% SDS, 0.01% bromophenol blue, 100 mM DTT) at 90°C. Next, genomic DNA was sheared by sonication, and the samples were boiled at 95°C for 5 minutes. The samples were then spun for 1 minute at 20,000 × g and run on a 10% SDS gel before transferring to nitrocellulose membranes (Bio-Rad) and blocking as previously described. ERK1/2 phosphorylation was detected by probing with anti-phospho-p44/42 (4370, Cell Signaling) and anti-tubulin (T6074, MilliporeSigma) primary antibodies and detected with fluorescent secondary antibodies (IRDye 800CW Donkey anti-Mouse and IRDye 680RD Goat Anti-Rabbit, LI-COR Biosciences) using LI-COR fluorescent imaging system. Next, the blots were stripped and reprobed with anti-total ERK (06–182, MilliporeSigma) and anti-tubulin and detected using the same secondary as previously described. Bands were quantified using ImageJ. The ratio of the phosphorylation of ERK to total ERK, each adjusted by corresponding tubulin density, was calculated for each lane to correct for differences in loading and staining efficiency. Values were normalized to the 0-minute time point on the respective membrane before averaging.

### Statistical Analyses

Statistical analyses were performed using SigmaPlot 11.0 software and methods described in the figure legends. Bar and symbol representation, along with error bars, are described in figure legends. Unless otherwise noted, scatter plots represent the average of three independent experiments measured in triplicate. For bar charts, the bars represent the average of three independent experiments, whereas the overlaid points report the values from the individual experiments measured in triplicate. All errors are reported as standard deviations. The responses to the CXCL12 titration experiments were fit to a sigmoidal dose–response model using SigmaPlot 11.0, and statistical significance was determined using the extra-sum-of-squares F test. One-way analysis of variance (ANOVA) followed by a Bonferroni *t* test, or unpaired *t* test in the case of comparing two samples, was used to determine statistical significance and *P* values for all other comparisons using SigmaPlot 11.0.

## Results

### GRKs Are Necessary for Efficient Scavenging of CXCL12 by ACKR3

Although GRK2 has been shown to contribute to ACKR3 internalization, *β*-arrestin recruitment, and chemokine scavenging, the relative importance of GRK2 versus other GRKs in these processes has not been determined (Saaber et al., 2019; Zarca et al., 2021). To assess the impact of GRKs on these ACKR3 functions, we first tested ACKR3 internalization and chemokine scavenging in HEK293A cells in which GRKs 2, 3, 5, and 6 were knocked out by CRISPR (ΔGRK2/3/5/6) (Pandey et al., 2021). ACKR3 internalization was monitored by bystander BRET between ACKR3 C-terminally tagged with luciferase (ACKR3_RlucII) and GFP fused to a CAAX domain (rGFP_CAAX) that anchors it to the plasma membrane (Namkung et al., 2016). In WT cells, CXCL12 induced a rapid decrease (from 100% to 66% ± 1%) in BRET signal corresponding to ACKR3 internalization (Fig. 1A). The internalization was almost eliminated in ΔGRK2/3/5/6 cells, with 97% ± 1% remaining BRET signal, indicating a critical role for GRKs.

We next determined the effect of the ΔGRK2/3/5/6 knockout on ACKR3 scavenging by ELISA quantification of CXCL12 remaining in the bulk media following incubation with cells transfected with a receptor or empty vector. Comparable receptor expression in the various cell lines was confirmed by luminescence from the C-terminal luciferase tag (Supplemental Fig. 1). In WT cells, ACKR3 efficiently removed 64% ± 3% of CXCL12 in comparison with non-ACKR3 expressing cells after overnight incubation (Fig. 1B). In the absence of GRKs, the chemokine uptake was severely impaired, with 75% ± 4% remaining in the bulk media. Interestingly, it appears that ACRK3 retains the capacity to clear approximately 20% of the added CXCL12 (Fig. 1B) even when internalization, as detected by BRET, is nearly eliminated (Fig. 1A). One possible explanation is that ACKR3 constitutively internalizes and recycles back to the plasma membrane, which has been previously shown to contribute to CXCL12 scavenging (Luker et al., 2010; Hoffmann et al., 2012). Constitutive internalization is not detected by BRET, which is only sensitive to changes following a perturbation of the equilibrium state (such as ligand-induced translocation of the receptor from the plasma membrane to the inside of the cell). Thus, to ascertain the impact of GRKs on ACKR3 constitutive internalization and recycling, we employed a “prelabel” flow cytometry experiment (Luker et al., 2010) instead of the BRET assay. In this experiment, surface receptors are first labeled with a nonconjugated anti-ACKR3 antibody at 4°C (a temperature that halts internalization) and then warmed to 37°C to allow for constitutive internalization and endocytic trafficking. The remaining original (prelabeled) receptor is detected by fluorescent secondary antibody staining, and the level of constitutive internalization is quantified by comparing the cells incubated at 37°C with control samples held at 4°C. In contrast to the ligand-stimulated internalization of the receptor detected by BRET, no difference was observed between WT and ΔGRK2/3/5/6 cells (Fig. 1C), consistent with constitutive trafficking occurring in the absence of GRKs. Note that in the remainder of the text, we interchangeably refer to ligand-stimulated BRET-detected internalization as “active internalization,” which, as demonstrated here, requires GRK-mediated phosphorylation, and constitutive internalization as “passive internalization,” which does not.

### GRK2 and GRK5 Differentially Regulate CXCL12-Mediated ***β***-Arrestin Recruitment and Internalization by ACKR3

**Fig. 2.**
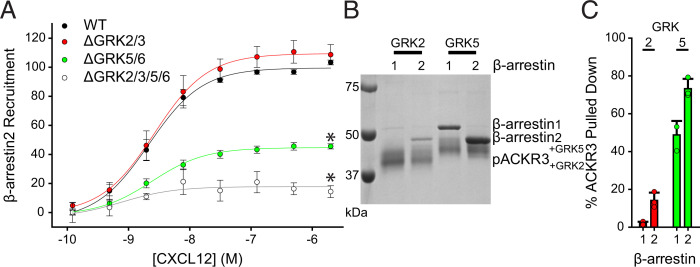
GRK5 plays a dominant role in ACKR3 phosphorylation and *β*-arrestin recruitment in HEK293A cells and with purified components. (A) Recruitment of GFP_ *β*arr2 to ACKR3_RlucII observed by BRET in ΔGRK HEK293A cell lines across a titration of CXCL12 concentrations. Responses are normalized to WT ACKR3 and are a composite of three independent experiments performed in triplicate. (B) Pulldowns of purified *β*-arrestin1 and 2 by purified ACKR3 phosphorylated in vitro by either GRK2 or GRK5. (C) Quantification of the amount of *β*-arrestin pulled down from three independent experiments. The amount of *β*-arrestin pulled down in C is presented as a percentage of the ACKR3 band density from the same experiment. Errors are reported as standard deviations and significance in A as determined by an extra-sum-of-squares F-test, *P < 0.001. EC_50_ values were not different among the tested conditions.

Having established the importance of GRKs to ACKR3 function, we next evaluated the relative contributions of specific GRKs. Using GFP10-tagged *β*-arrestin2 (GFP_*β*arr2) and ACKR3_RlucII, *β*-arrestin2 recruitment to ACKR3 was monitored by BRET in GRK2/3 and GRK5/6 CRISPR-knockout cells (ΔGRK2/3 and ΔGRK5/6 cells, respectively) (Pandey et al., 2021). Recruitment in ΔGRK5/6 cells was reduced to 45% ± 2% of the WT maximum (Fig. 2A). In contrast, recruitment in ΔGRK2/3 cells was identical to the WT receptor. As expected, *β*-arrestin recruitment in ΔGRK2/3/5/6 cells was nearly abolished. Prior experiments individually overexpressing the four GRKs showed that only GRK2 and GRK5 phosphorylate ACKR3 (Saaber et al., 2019), which is supported by a lack of observed CXCL12-promoted recruitment of GRK6 to ACKR3 (Zarca et al., 2021), although GRK3 results were less consistent between assays (Saaber et al., 2019; Zarca et al., 2021). Therefore, although we cannot fully discount the contributions of the other GRKs, we attribute the results using the ΔGRK2/3 and ΔGRK5/6 cells primarily to the loss of GRK2 and GRK5, respectively. These results were corroborated by in vitro pulldowns of purified ACKR3-*β*-arrestin complexes phosphorylated with each kinase (Fig. 2B). The more extensive upward shift of the receptor band for GRK5-phosphorylated ACKR3 suggests greater phosphorylation compared to GRK2 under similar conditions. Additionally, the pulldowns of GRK5 phosphorylated ACKR3 revealed that approximately 49% ± 7% and 73% ± 5% of the receptor was complexed with *β*-arrestin1 and 2, respectively (Fig. 2C), whereas only 14% ± 4% of the receptor was complexed with *β*-arrestin2, and almost no *β*-arrestin1 was detected in the pulldowns of GRK2 phosphorylated ACKR3. Together, the in-cell and in vitro methods point to the same conclusion: *β*-arrestin recruitment to ACKR3 is dominated by GRK5 phosphorylation under our conditions.

**Fig. 3.**
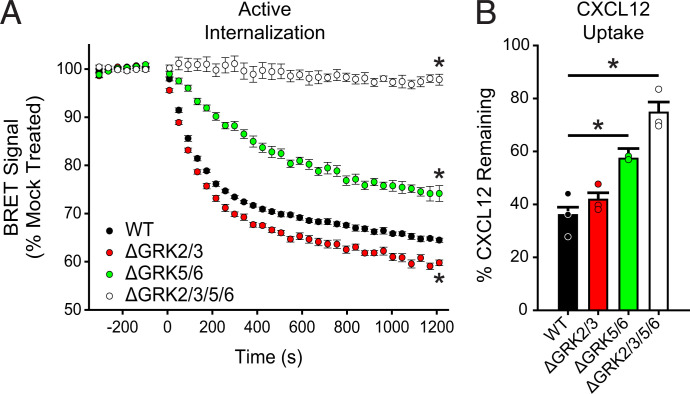
ACKR3 requires GRK5 for efficient active internalization and CXCL12 scavenging in HEK293A cells. (A) Active CXCL12-promoted internalization of ACKR3 monitored by BRET following stimulation with 100 nM CXCL12 in ΔGRK HEK293A cell lines at 37°C. (B) CXCL12 uptake by ACKR3 expressed in ΔGRK cell lines quantified by ELISA and presented as a percentage of identical cells transfected with empty vector. Data are composites of three independent experiments measured in triplicate, errors are reported as standard deviations, and statistical significance was determined by one-way ANOVA followed by a Bonferroni test. *P < 0.001. Data for WT and ΔGRK2/3/5/6 cells are repeated from Fig. 1 for comparison.

Because GRKs are necessary for CXCL12-mediated internalization of ACKR3, we also tested the impact of GRK2/3 or GRK5/6 phosphorylation on ligand-induced membrane trafficking. ACKR3 on the plasma membrane was once again monitored by BRET between ACKR3_RlucII and rGFP_CAAX. Similar to *β*-arrestin recruitment, active internalization of ACKR3 in ΔGRK5/6 cells was impaired (∼25% BRET loss) compared with WT cells (∼35% BRET loss) (Fig. 3A). In contrast, the internalization was slightly enhanced (∼5%) in the ΔGRK2/3 cells. Together with the lack of effect on *β*-arrestin recruitment, these results suggest little impact of GRK2 phosphorylation when GRK5 is present in these cells, consistent with recent reporting (Sarma et al., 2022).

Finally, we tested how specific GRKs alter CXCL12 scavenging. ACKR3 only removed 43% ± 4% of CXCL12 when expressed in ΔGRK5/6 cells compared to more than 60% in WT cells. In contrast, CXCL12 scavenging in ΔGRK2/3 cells was indistinguishable from WT (Fig. 3B), again consistent with little contribution of GRK2 when GRK5 is present. However, the ΔGRK2/3/5/6 cells showed even less scavenging than the ΔGRK5/6 cells (∼20% scavenged). Moreover, all tested ACKR3 functions showed a greater decrease in the ΔGRK2/3/5/6 cells compared to the ΔGRK5/6 cells, suggesting that GRK2 and 5 may synergize.

### Mutation of ACKR3 Phosphorylation Site Clusters Correlate with Effects of GRK Knockout Cells

**Fig. 4.**
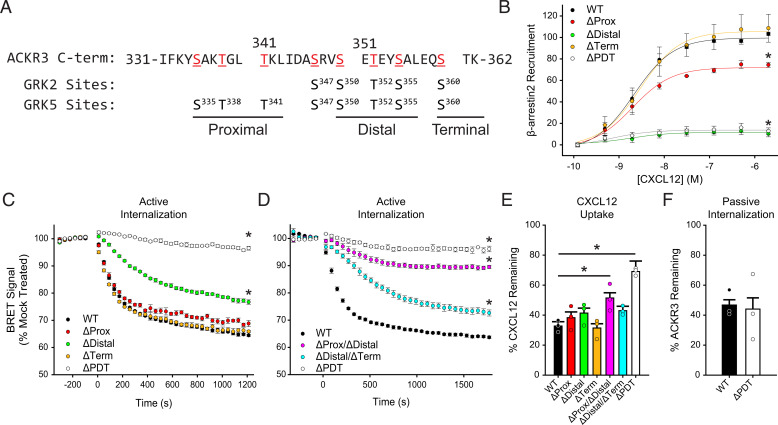
Specific phosphorylation motifs differently contribute to CXCL12 responses by ACKR3 in HEK293A cells. (A) ACKR3 was phosphorylated by either GRK2 or GRK5 in vitro, and the specific sites of modification were determined by mass spectrometry. The detected phosphorylation sites are highlighted in red. Phosphorylated positions in the ACKR3 C-terminus were divided into three clusters and replaced by alanine to produce ΔProx, ΔDistal, ΔTerm, and ΔPDT receptor constructs. (B) Recruitment of GFP_*β*arr2 to phosphorylation-deficient ACKR3 constructs expressed in WT HEK293A cells and tested across a titration of CXCL12 and detected by BRET. Values represent three independent experiments performed in triplicate and normalized to WT ACKR3 recruitment. (C and D) Active internalization of individual (C) and multiple (D) phosphorylation cluster substitutions tracked by the loss of BRET between ACKR3_RlucII and rGFP_CAAX after CXCL12 addition. (E) CXCL12 uptake by phosphorylation deficient ACKR3 constructs, measured by remaining chemokine and compared with cells transfected with empty vector. (F) Passive agonist-independent internalization of the triple phosphorylation substitution observed by the prelabeled flow cytometry experiment. WT ACKR3 data in B, C, E, and F were repeated from Figs. 1 and 2A for comparison. All error bars represent standard deviations, and statistical significance was determined by (B) the extra sum of squares F-test and (C, D, and E) one-way ANOVA followed by a Bonferroni test. *P < 0.001.

The different functional responses of GRK2 and GRK5 phosphorylated ACKR3 suggest that the kinases introduce distinct phosphorylation barcodes and/or levels of phosphorylation. Accordingly, mass spectrometry of purified and in vitro phosphorylated ACKR3 was used to identify the positions modified by each kinase (Supplemental Fig. 2; Supplemental Table 1; Supplemental Data Files). As shown in Fig. 4a, both GRK2 and GRK5 preferentially phosphorylate distal positions of the receptor C-terminus (S347/S350/T352/S355/S360), which were previously shown to be important for *β*-arrestin binding to ACKR3 (Zarca et al., 2021) and efficient chemokine scavenging (Hoffmann et al., 2012). GRK5 also preferentially phosphorylated proximal sites (S335/T338/T341). To further explore the role of these sites, as well as the terminal cluster S360/T361, we mutated to alanine all Ser/Thr residues in each of the three clusters, as indicated in Fig. 4A.

Mutation of the distal sites (ΔDistal) completely eliminated *β*-arrestin2 recruitment (Fig. 4B) (Zarca et al., 2021). The fact that the distal mutation had a greater impact on recruitment than loss of GRK5/6 or GRK2/3, even though both GRKs phosphorylate the distal sites, is likely due to compensation by the remaining kinases in the knockout cells. Mutation of the terminal phosphate sites (ΔTerm) displayed similar *β*-arrestin recruitment to WT ACKR3. Partial impairment exhibited by the proximal site mutant (ΔProx) was similar to that of the ΔGRK5/6 cells (72.3% ± 2.4% of the WT response). Together, the data suggest that the relative importance of the phosphorylation clusters for *β*-arrestin binding is distal > proximal > terminal, giving an explanation for why GRK5 appears to phosphorylate ACKR3 more efficiently than GRK2 and dominates *β*-arrestin recruitment (Fig. 2). Additionally, and as discussed in the following text, GRK5 does not require G*βγ* for efficient phosphorylation, whereas GRK2 does (Pitcher et al., 1992). Because ACKR3 does not activate G proteins (Rajagopal et al., 2010), the contribution of GRK2 is predictably limited.

We also investigated the effects of the phosphorylation cluster mutations on CXCL12-induced active internalization of ACKR3 by BRET. The ΔDistal mutant receptor showed significantly reduced trafficking (78% ± 0.7% remaining BRET), whereas the ΔTerm and ΔProx constructs were similar to WT ACKR3 (66% ± 1% remaining) (Fig. 4C). The GRK5-specific cluster construct (ΔProx/ΔDistal) showed even further impairment of the internalization (90% ± 0.6% of unstimulated BRET) (Fig. 4D) while the triple cluster knockout ΔPDT mutant showed no agonist-induced, active internalization. By contrast, ΔDistal/ΔTerm (Fig. 4D) showed similar internalization as the ΔDistal construct (Fig. 4C). These results suggest that, as for arrestin recruitment, the distal phosphorylation sites are most important for efficient active internalization. Mutations of the proximal and terminal sites only impaired trafficking when combined with the distal motif, with the proximal being more impactful than the terminal sites.

In contrast to agonist-mediated, active ACKR3 internalization, scavenging of CXCL12 was not affected by mutation of the individual phosphorylation clusters, including the ΔDistal mutation (Fig. 4E). Only the ΔPDT mutant and the ΔProx/ΔDistal mutant showed significantly reduced chemokine uptake, only removing 30% ± 7% and 49% ± 4% of the chemokine, respectively. The amount of CXCL12 removed by the ΔPDT and ΔProx/ΔDistal mutants was similar to the chemokine uptake by WT ACKR3 in ΔGRK2/3/5/6 and ΔGRK5/6 cells, respectively, again indicating the dominance of GRK5.

Finally, we also examined the effects of the phosphorylation mutations on constitutive (passive) internalization. As with the ΔGRK2/3/5/6 cells, the triple cluster phosphorylation mutation construct (ΔPDT) had no effect (Fig. 4F). The independence of constitutive internalization and dependence of scavenging on ACKR3 phosphorylation is consistent with a previous study (Saaber et al., 2019). Altogether, our data suggest that chemokine scavenging by ACKR3 is mediated by both a phosphorylation-dependent internalization process (dominated by GRK5 in HEK293A cells) and, to a lesser extent, by a phosphorylation-independent constitutive internalization process. The phosphorylation dependence led us to consider the role of *β*-arrestin.

### ***β***-Arrestins Are Dispensable for ACKR3 Internalization and Scavenging

**Fig. 5.**
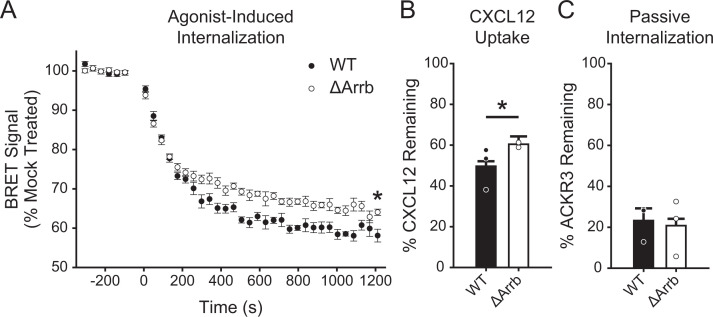
*β*-arrestins are dispensable for ACKR3 internalization and CXCL12 scavenging in HEK293 cells. (A) CXCL12-mediated, active internalization in WT and ΔArrb HEK293 cells, measured by decreasing BRET between rGFP_CAAX and ACKR3_RlucII. Note that the cellular background for ΔArrb is HEK293, which leads to slightly different responses compared with the HEK293A cells used in all other figures. (B) Chemokine uptake by WT ACKR3 in ΔArrb and WT HEK293 cells. The remaining chemokine was quantified by ELISA and compared with non-ACKR3 expressing cells. (C) ACKR3 agonist-independent, passive internalization in WT and ΔArrb HEK293 cells. Data presented are the composite of three independent experiments measured in triplicate, and errors represent standard deviations. Statistical significance was determined by an unpaired *t* test. *P < 0.05.

Phosphorylation of GPCRs triggers their interaction with *β*-arrestins, which then often facilitate receptor internalization. Because internalization is a key requirement of scavenging and ACKR3 scavenging is dependent on phosphorylation, a logical hypothesis would be that *β*-arrestins mediate the process. However, the role of *β*-arrestins in scavenging has been controversial, with some reports suggesting arrestins are essential for ACKR3 internalization and scavenging (Luker et al., 2010; Canals et al., 2012) and others suggesting they are dispensable (Montpas et al., 2018; Saaber et al., 2019; Zarca et al., 2021). To assess the role of *β*-arrestin in our system, we conducted experiments in *β*-arrestin1/2-knockout (ΔArrb) HEK293 cells (Milligan and Inoue 2018). As shown in Fig. 5, CXCL12-mediated active internalization (Fig. 5A) and CXCL12 scavenging (Fig. 5B) show little difference in ΔArrb cells compared to WT cells. Likewise, constitutive (passive) internalization was not impacted by the loss of arrestins (Fig. 5C). Thus, although *β*-arrestin is recruited to the receptor, it has only a minor effect on agonist-induced internalization and chemokine uptake, suggesting a role in a yet-to-be-identified function.

### G***βγ*** enhances GRK2-Mediated Responses of ACKR3

**Fig. 6.**
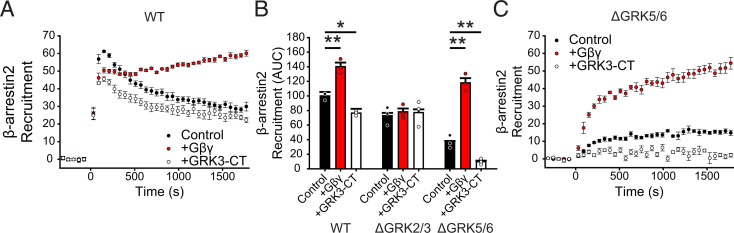
GRK2 phosphorylation of ACKR3 is enhanced by expression of G*βγ* in HEK293A cells. (A) GFP_*β*arr2 recruitment to ACKR3_RlucII measured by BRET after 100 nM CXCL12 addition at 0 minute in WT HEK293A cells co-transfected with G*βγ* or GRK3-CT. (B) Quantification of Barr2 recruitment in WT, ΔGRK2/3, and ΔGRK5/6 cells with co-transfection with G*βγ* or GRK3-CT by integration of the area under the BRET curves after chemokine addition. Areas are normalized to WT ACKR3 recruitment in WT cells without additional treatment. (C) Recruitment of *β*-arrestin2 to ACKR3 in ΔGRK5/6 cells with G*βγ* and GRK3-CT co-expression. Data represent three independent experiments measured in triplicate, and individual experiments are presented as points in (B). Errors are reported as standard deviation, and statistical significance was determined by one-way ANOVA followed by a Bonferroni test. *P < 0.01, **P < 0.001.

Although GRK5 was the dominant kinase in our HEK293A system, other reports suggest that GRK2 plays an important role in driving ACKR3 responses in HEK293 cells and primary neurons (Saaber et al., 2019; Zarca et al., 2021). One explanation for the observed dominance of GRK5 in our experiments may be the inability of ACKR3 to activate G proteins and release G*βγ* (Digby et al., 2006; Rajagopal et al., 2010; Yen et al., 2022) because G*βγ* is required for GRK2 membrane recruitment and efficient receptor phosphorylation (Pitcher et al., 1992; Koch et al., 1993; Lodowski et al., 2005). In contrast, GRK5 is constitutively associated with membrane phospholipids and does not bind or require G*βγ* (Kunapuli et al., 1994; Pitcher et al., 1996). To test the hypothesis that GRK2 phosphorylation of ACKR3 is enhanced by increasing free G*βγ*, we used *β*-arrestin recruitment to ACKR3 as an indirect measure of the phosphorylation status of the receptor. In WT cells co-transfected with G*β*_1_*γ*_2_ along with ACKR3_RlucII and *β*arr2_GFP, *β*-arrestin recruitment was 140% ± 6% (area under the curve) compared to cells without additional G*βγ* (Fig. 6, A and B). The effect of G*βγ* addition in ΔGRK5/6 cells was even greater, increasing from 37% ± 2% of WT without extra G*βγ* to 120% ± 6% with the co-transfection (Fig. 6, B and C). Overexpression of GRK3-CT, which inhibits GRK2/3 phosphorylation by sequestration of free G*βγ* (Koch et al., 1993; Boekhoff et al., 1994; Koch et al., 1994), suppressed *β*-arrestin recruitment in WT cells (77% ± 6% of WT) (Fig. 6A) and nearly eliminated the interaction in ΔGRK5/6 cells (9.8% ± 1% of WT) (Fig. 6C). Neither overexpression of G*βγ* nor GRK3-CT had an effect on *β*-arrestin recruitment in ΔGRK2/3 cells, indicating that these treatments were specific to GRK2 and not GRK5 (Fig. 6B).

**Fig. 7.**
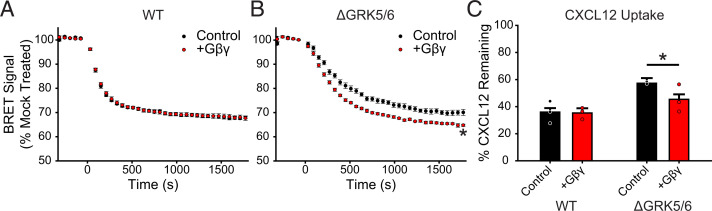
G*βγ* co-transfection enhances ACKR3 internalization and CXCL12 scavenging but only in the absence of GRK5/6 in HEK293A cells. (A and B) ACKR3_RlucII active internalization was tracked by rGFP_CAAX BRET with and without co-transfection of G*βγ* subunits in WT (A) and ΔGRK5/6 (B) cells following addition of 100 nM CXCL12. Plots are the average of three independent experiments measured in triplicate. (C) CXCL12 uptake in WT and ΔGRK5/6 cells with G*βγ* co-transfection. Data from cells without extra G*βγ* are repeated here from Fig. 3 for comparison. Errors were reported as standard deviations, and statistical significance was determined by one-way ANOVA followed by a Bonferroni test. *P < 0.001.

We next tested whether G*βγ* enhanced GRK2 activity also improves ACKR3 active internalization and chemokine scavenging. In WT cells, G*βγ* co-expression had no effect on agonist-induced active ACKR3 internalization (Fig. 7A); however, a modest increase in internalization was observed in ΔGRK5/6 cells with added G*βγ* (71% ± 0.9% remaining BRET without G*βγ* to 66% ± 0.6% with the co-expression) (Fig. 7B). Likewise, CXCL12 scavenging was significantly enhanced in ΔGRK5/6 cells with added G*βγ*, but not in WT cells (Fig. 7C). Together these results are consistent with efficient active internalization and scavenging by ACKR3 phosphorylated by GRK5. However, in the absence of GRK5, GRK2 plays a more important role and requires free G*βγ* for efficient activity.

**Fig. 8.**
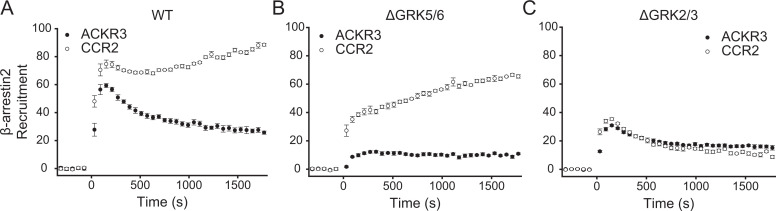
*β*-arrestin2 recruitment to the canonical GPCR CCR2 is mediated primarily by GRK2/3. A-C) GFP_*β*arr2 recruitment to ACKR3_RlucII or CCR2_rlucII after stimulation with either 100 nM CXCL12 or CCL2, respectively, was tracked by BRET in WT (A), ΔGRK5/6 (B), and ΔGRK2/3 (C) cells. Data are the average of three independent experiments performed in triplicate, and errors reflect standard deviations.

Finally, to ascertain that the observed dominance of GRK5 was not a consequence of insufficient GRK2, we performed the BRET-based *β*-arrestin2 recruitment experiment with CCR2, a CCKR, which is known to be phosphorylated by GRK2/3 (Aragay et al., 1998). CCL2-activated CCR2 showed robust association with *β*-arrestin2 in WT and ΔGRK5/6 cells, indicating ample phosphorylation by GRK2/3 (Fig. 8, A and B). In fact, the recruitment profiles to CCR2 were similar to those of ACKR3 in ΔGRK5/6 cells when G*βγ* was present, including a prolonged association of the receptors with *β*-arrestin2 (Figs. 6 and 8). In ΔGRK2/3 cells, *β*-arrestin2 recruitment to the two receptors was also nearly identical (Fig. 8C). Together, these data suggest that the observed dominance of GRK5 in ACKR3 phosphorylation is largely due to a lack of free G*βγ* and not a limitation of GRK2 expression or kinase preference for ACKR3.

### Co-Activation of CXCR4 Enhances GRK2-Mediated Responses of ACKR3

**Fig. 9.**
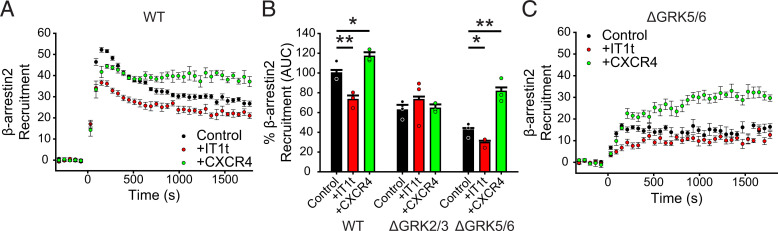
Concurrent CXCR4 activation increases ACKR3 phosphorylation by GRK2/3. (A) GFP_*β*arr2 recruitment to ACKR3_RlucII after 100 nM CXCL12 addition in WT cells treated with 100 *µ*M IT1t for 45 minutes before the experiment or co-transfected with additional CXCR4 DNA. Individual points are the average of three experiments measured in triplicate. (B) Area under the curve analysis of GFP_*β*arr2 recruitment time courses after CXCL12 addition normalized to WT ACKR3_RlucII in WT cells without treatment. (C) Recruitment time courses of GFP_*β*arr2 to ACKR3_RlucII by BRET in ΔGRK5/6 cells treated identically to the WT cells described in (A). Errors were represented by standard deviation, and statistical significance was determined by one-way ANOVA followed by a Bonferroni test. *P < 0.005, **P < 0.001.

Because ACKR3 does not activate G proteins, we hypothesized that there must be another source of G*βγ* for GRK2 to contribute to ACKR3 phosphorylation in WT and ΔGRK5/6 cells. Low endogenous levels of CXCR4 have been observed in HEK293 cells (Kufareva et al., 2014). Accordingly, we first tested whether the small molecule CXCR4 antagonist, IT1t (Thoma et al., 2008), would suppress GRK2 phosphorylation and *β*-arrestin recruitment to ACKR3 by virtue of blocking G*βγ* release by CXCL12-activated CXCR4. As expected, IT1t had no effect in the ΔGRK2/3 cells. However, there was a significant decrease in *β*-arrestin recruitment to ACKR3 with IT1t treatment in WT (73% ± 4% of untreated WT) and ΔGRK5/6 cells (42% ± 2% untreated compared with 30% ± 2% treated) (Fig. 9, A and B), consistent with the presence and contribution of endogenous CXCR4. As a side note, ACKR3 has been widely reported to signal through *β*-arrestin, resulting in phosphorylation of ERK1/2 and AKT (Rajagopal et al., 2010; Decaillot et al., 2011; Lipfert et al., 2013), although more recent studies have called these observations into question (Saaber et al., 2019). As shown in Supplemental Fig. 3, we observed that CXCL12-mediated phosphorylation of ERK is inhibited by IT1t, suggesting that it is also due to a small level of endogenous CXCR4 and not ACKR3.

Finally, we also tested whether additional exogenous expression of CXCR4 could enhance GRK2-mediated ACKR3 activities. As shown in Fig. 9, A and B, the addition of CXCR4 slightly increased CXCL12-mediated *β*-arrestin2 recruitment to ACKR3 in WT cells to 120% ± 4% control conditions. However, in ΔGRK5/6 cells, exogenously added CXCR4 doubled the recruitment BRET from 42% ± 2% to 81% ± 2%, presumably by liberating G*βγ* and enhancing GRK2 activity (Fig. 9, B and C). The ability of CXCR4 activation to regulate the activity of ACKR3 may be the source of some of the discrepancies related to ACKR3 function reported in the literature (Rajagopal et al., 2010; Luker et al., 2012; Odemis et al., 2012; Montpas et al., 2018; Fumagalli et al., 2020). It also provides a means of crosstalk between the two receptors.

## Discussion

**Fig. 10.**
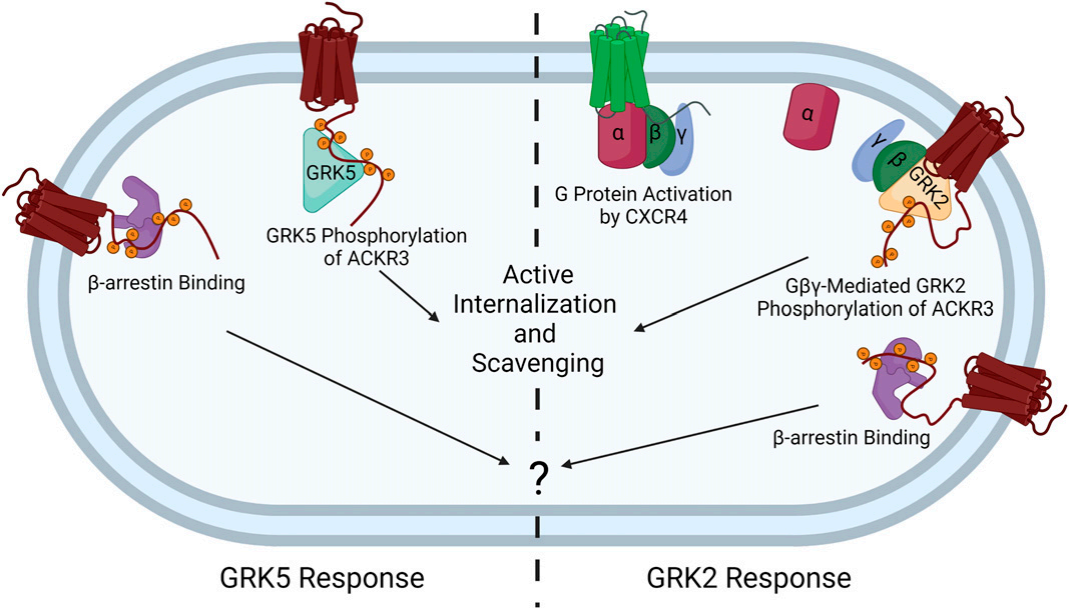
ACKR3 GRK specificity is dependent on whether CXCR4 is co-activated. When expressed alone, the atypical receptor is primarily phosphorylated by GRK5, which drives agonist-mediated internalization and scavenging. In the context of CXCR4 co-expression, the co-activation of CXCR4 activates heterotrimeric G proteins and releases G*βγ*, which recruits GRK2 and mediates phosphorylation of both receptors. This allows for ACKR3 to sense the activation of CXCR4 and then enhance the scavenging decoy responses of ACKR3. This image does not include the passive constitutive internalization and scavenging pathway. Image created with BioRender.

Both GRK2 and GRK5 have been reported to phosphorylate the C-terminus of ACKR3 (Canals et al., 2012; Saaber et al., 2019), a post-translational modification that is counterbalanced by ubiquitination in regulating ACKR3 levels and scavenging capacity (Lau et al., 2020; Wong et al., 2020). However, only GRK2 has been reported to promote *β*-arrestin interaction, receptor internalization, and chemokine uptake (Saaber et al., 2019; Zarca et al., 2021), whereas the relative contribution of GRK5 has not been systematically studied. Here we undertook a comparative study of these two ubiquitously expressed kinases to understand their phosphorylation patterns, their relative phosphorylation efficiencies, and their role in ACKR3 activities. We also sought to determine how GRK2 regulates ACKR3, given that GRK2 depends on G*βγ* for efficient phosphorylation, yet most studies suggest ACKR3 does not activate heterotrimeric G proteins necessary for G*βγ* release. Because GRK5 functions in a G protein–independent manner, it is expected to more efficiently phosphorylate ACKR3 than GRK2, all else being equal. This turned out to be the case in our studies using HEK293A cells, where GRK5 dominated ACKR3 phosphorylation over GRK2, consistent with recent reports (Sarma et al., 2022). Phosphorylation by GRK2 was more limited and observed most strongly as a consequence of CXCR4 and ACKR3 co-stimulation by CXCL12. Additionally, supplementation of CXCR4 increased phosphorylation of ACKR3 by GRK2, whereas IT1t antagonism of CXCR4 suppressed it nearly completely. These results suggest a means for ACKR3 to “sense” CXCR4 activation and respond by increasing chemokine uptake (Fig. 10). By including a role for CXCR4 in the phosphorylation process, this mechanism expands the competing phosphorylation-ubiquitination feedback regulation of ACKR3 by extracellular CXCL12 levels proposed by Wong et al. (Wong et al., 2020). Excess CXCL12 activates additional CXCR4, which in turn promotes chemokine scavenging by ACKR3. ACKR3/CXCR4 crosstalk would obviously require that the receptors are expressed in the same cell, as observed in cortical interneurons (Saaber et al., 2019). Alternatively, in cells with low CXCR4 expression, such as our HEK293A cells, ACKR3 phosphorylation requires a G*βγ*-independent kinase like GRK5. The results also suggest that the phosphorylation of ACKR3 by GRK2 reported in earlier studies (Saaber et al., 2019; Zarca et al., 2021) is likely promoted by endogenous CXCR4.

Recently, GPCRs have been categorized by GRK specificity into GRK2/3 and GRK2/3/5/6 subclasses (Drube et al., 2022). Under this paradigm, CCR2, for example, would fit into the GRK2/3 classification (Fig. 8), while ACKR3 would fall into the GRK2/3/5/6 class due to its strong GRK5 bias. However, the presence of active G proteins shifts ACKR3 GRK specificity to be more similar to CCR2, suggesting that ACKR3 GRK preference is not mediated by kinase interactions but by G proteins. Therefore, an obvious question is whether similar GRK discrimination is observed for other receptor systems that are biased away from G proteins. Consistent with the bias model, Pandey and colleagues demonstrated that the atypical receptors C5a receptor subtype 2 and ACKR2 both show a decrease in *β*-arrestin recruitment in ΔGRK5/6 cells, whereas ΔGRK2/3 cells have little to no impact (Pandey et al., 2021). In contrast, canonical, G protein–activating receptors like CCR2 (Fig. 8) (Aragay et al., 1998; Shroka et al., 2023) and *µ*-opioid receptors (Møller et al., 2020) are both preferentially phosphorylated by GRK2/3. The behavior also appears to extend to biased ligands. When the angiotensin II type 1 receptor is stimulated by the neutral agonist angiotensin II, phosphorylation is mediated by GRK2/3. However, the arrestin-biased ligand TRV027 completely shifts this dependence to GRK5/6, consistent with G protein activation mediating GRK discrimination (Kawakami et al., 2022). In an exception to this paradigm, ACKR4, which does not couple to G proteins, exclusively associates with G*βγ*-regulated GRK2 and GRK3 (Matti et al., 2020). This result suggests that a novel interaction may promote GRK2/3 phosphorylation that is distinct from the canonical mechanism or that another CCKR responds to the ACKR4 ligands CCL19, CCL21, and CCL25 and supplies G*βγ*. Although it is tempting to propose this GRK regulation model as a general feature of arrestin-biased systems, more detailed studies are needed to fully understand how extensively it applies and to explain exceptions such as ACKR4.

The availability of GRKs has been proposed as a mechanism for modulating GPCR function in different cellular systems (Matthees et al., 2021). Functional differences due to GRK phosphorylation have been demonstrated for many receptors, including CXCR4 (Busillo et al., 2010), *β*2 adrenergic receptor (Nobles et al., 2011), and others (Møller et al., 2020; Pandey et al., 2021; Drube et al., 2022; Kawakami et al., 2022). Our results suggest a unique twist where the different GRKs alter the regulation of a given function—in this case, chemokine scavenging by ACKR3—with potential consequences on the activity of another receptor. Specifically, when the receptor is expressed alone, GRK5 drives chemokine-mediated phosphorylation of ACKR3. However, in the presence of CXCR4, phosphorylation by GRK2 is enhanced, which in turn may limit the availability of CXCL12 to CXCR4, leading to effects on CXCR4 signaling, desensitization, and degradation (Bhandari et al., 2007; Saaber et al., 2019). ACKR3 also shares agonists with CXCR3 (CXCL11) (Burns et al., 2006) and opioid receptors (opioid peptides) (Meyrath et al., 2020); therefore, it is conceivable that GRK-based cross-regulation between these GPCRs and ACKR3 could occur. Whether phosphorylation by GRK2 versus GRK5 also leads to differences in ACKR3 functional responses remains to be determined. However, it is notable that GRK2-mediated phosphorylation leads to different phosphorylation patterns on the ACKR3 C-terminus and more persistent complexes with *β*-arrestin than GRK5 (Figs. 4 and 6).

Despite the requirement of phosphorylation for agonist-induced internalization of ACKR3, scavenging was not completely abolished in the ΔGRK2/3/5/6 cells and when all C-terminal phosphorylation sites in ACKR3 were removed. Rather, low-level (20%–30%) chemokine uptake persisted in both cases. Constitutive internalization and recycling of ACKR3 were also fully retained under these conditions. These data suggest that in addition to the active, agonist-induced phosphorylation-dependent pathway, an alternative passive, constitutive phosphorylation-independent mechanism exists for transporting chemokine into cells, a mechanism that is not unprecedented. For example, we recently demonstrated that scavenging by CCR2, a “dual function” chemokine receptor that also couples to G proteins, continues in the absence of phosphorylation (Shroka et al., 2023). Furthermore, the low-level uptake of chemokine by phospho-deficient ACKR3 may explain observations that whereas ACKR3 knockout mice are embryonically lethal (Sierro et al., 2007), mice expressing a phosphorylation-deficient ACKR3 mutant are viable (Saaber et al., 2019). Nevertheless, the phospho-deficient ACKR3 mice showed defects in neuronal CXCR4 expression and responsiveness to CXCL12 as well as cell migration, suggesting that constitutive scavenging is unable to fully regulate chemokine levels (Saaber et al., 2019). Along these lines, the zebrafish primordium expressing phospho-deficient ACKR3 migrated faster than the primordium without the atypical receptor, albeit with lower efficiency compared to WT ACKR3 (Lau et al., 2020; Wong et al., 2020). Together, these data suggest that constitutive internalization and recycling of ACKR3 may effectively regulate low levels of chemokine even in the absence of phosphorylation, but receptor phosphorylation is required to accommodate chemokine surges. Indeed, Wong et al. showed support for this hypothesis by demonstrating that phosphorylation increases ACKR3 levels and scavenging efficiency by enhancing plasma membrane recycling rather than degradation (Wong et al., 2020).

Due to the *β*-arrestin bias of ACKR3, coupled with the role of *β*-arrestin in regulating the internalization of many GPCRs, the expectation was that scavenging by ACKR3 would occur in a *β*-arrestin–dependent manner. However, the role of *β*-arrestin in scavenging has been controversial, with some reports involving HEK293 and mouse embryonic fibroblasts indicating it is necessary for ACKR3 internalization and chemokine scavenging (Luker et al., 2010; Canals et al., 2012), and others involving mouse neurons and HEK293 cells showing no impact (Montpas et al., 2018; Saaber et al., 2019; Zarca et al., 2021). Our data also suggest that constitutive and agonist-induced internalization, as well as scavenging of CXCL12, occurs largely independent of *β*-arrestin. ACKR3 has been shown to signal through ERK/MapK/AKT pathways via *β*-arrestins (Rajagopal et al., 2010; Decaillot et al., 2011; Lipfert et al., 2013; Ishizuka et al., 2021), although this appears to be limited to cells with currently active heterotrimeric G protein signaling (Grundmann et al., 2018). Likewise, we demonstrate that ERK/MapK phosphorylation in our HEK293 cells is CXCR4 dependent (Supplemental Fig. 3). Because ACKR3 readily recruits *β*-arrestin in response to almost every ligand (Yen et al., 2022), arrestins likely play critical roles in some common ACKR3-mediated process, but that role remains a mystery.

In conclusion, our study reveals differences in ACKR3 regulation in response to the activity of GRK2 and GRK5. GRK5 dominates when ACKR3 binds CXCL12 in isolation, whereas GRK2 phosphorylates ACKR3 when CXCR4 and ACKR3 are co-stimulated by CXCL12, providing a readout of CXCR4 activation status, increasing CXCL12 scavenging, and possibly modulating CXCR4 responses and trafficking. Because ACKR3 is also a receptor for the chemokine CXCL11, similar crosstalk may exist in cells co-expressing CXCR3. Crosstalk with other receptors that do not share a common ligand is also conceivable.

## Abbreviations

ACKR: atypical chemokine receptor
ACKR3: atypical chemokine receptor 3
BRET: bioluminescence resonance energy transfer
CCKR: canonical chemokine receptor
CCR2: CC chemokine receptor 2
CHS: cholesteryl hemisuccinate
CXCR4: CXC chemokine receptor 4
CXCR3: CXC chemokine receptor 3
DMEM: Dulbecco’s modified Eagle’s media
DTT: dithiothreitol
ERK: extracellular signal-regulated kinase
FACS: fluorescence-activated cell sorter
GPCR: G protein-coupled receptor
GRK: GPCR kinase
GRK3-CT: GRK3-C-terminus
IPTG: isopropyl *β*-d-thiogalactopyranoside
IT1t: isothiourea-1t
LMNG: lauryl maltose neop entyl glycol
MOPS: 4-morpholinepropanesulfonic acid
PMSF: phenylmethylsulfonyl fluoride
rGFP: *Renilla* green fluorescent protein
RlucII: *Renilla* luciferase II
TCEP: Tris (2-carboxyethyl) phosphine
WT: wild-type
